# Plant Growth-Promoting Rhizobacteria Eliminate the Effect of Drought Stress in Plants: A Review

**DOI:** 10.3389/fpls.2022.875774

**Published:** 2022-08-11

**Authors:** Hafiz Muhammad Ahmad, Sajid Fiaz, Sumaira Hafeez, Sadaf Zahra, Adnan Noor Shah, Bushra Gul, Omar Aziz, Mahmood-Ur-Rahman, Ali Fakhar, Mazhar Rafique, Yinglong Chen, Seung Hwan Yang, Xiukang Wang

**Affiliations:** ^1^Department of Bioinformatics and Biotechnology, Government College University, Faisalabad, Pakistan; ^2^Department of Plant Breeding and Genetics, The University of Haripur, Haripur, Pakistan; ^3^Department of Plant Breeding and Molecular Genetics, University of Poonch, Rawalakot, Pakistan; ^4^Department of Agricultural Engineering, Khwaja Fareed University of Engineering and Information Technology, Rahim Yar Khan, Pakistan; ^5^Department of Biosciences, University of Wah, Wah, Pakistan; ^6^Department of Soil and Environmental Science, University of Agriculture, Faisalabad, Pakistan; ^7^Department of Soil and Climate Change, The University of Haripur, Haripur, Pakistan; ^8^School of Agriculture and Environment, UWA Institute of Agriculture, University of Western Australia, Perth, WA, Australia; ^9^Department of Biotechnology, Chonnam National University, Yeosu, South Korea; ^10^College of Life Sciences, Yan’an University, Yan’an, China

**Keywords:** soil microbes, microbiome, drought, endosphere, rhizosphere

## Abstract

Plants evolve diverse mechanisms to eliminate the drastic effect of biotic and abiotic stresses. Drought is the most hazardous abiotic stress causing huge losses to crop yield worldwide. Osmotic stress decreases relative water and chlorophyll content and increases the accumulation of osmolytes, epicuticular wax content, antioxidant enzymatic activities, reactive oxygen species, secondary metabolites, membrane lipid peroxidation, and abscisic acid. Plant growth-promoting rhizobacteria (PGPR) eliminate the effect of drought stress by altering root morphology, regulating the stress-responsive genes, producing phytohormones, osmolytes, siderophores, volatile organic compounds, and exopolysaccharides, and improving the 1-aminocyclopropane-1-carboxylate deaminase activities. The use of PGPR is an alternative approach to traditional breeding and biotechnology for enhancing crop productivity. Hence, that can promote drought tolerance in important agricultural crops and could be used to minimize crop losses under limited water conditions. This review deals with recent progress on the use of PGPR to eliminate the harmful effects of drought stress in traditional agriculture crops.

## Introduction

Water is the most indispensable requirement for the growth and development of agricultural crops (Javed et al., 2016). The term drought generally implies a lower supply of irrigation water than the demand (Ali et al., 2016). Osmotic stress has been ranked as the most harmful environmental stress factor worldwide (Marchin et al., 2020). Changing climatic conditions have triggered drought stress in several parts of the world (Javed et al., 2016; Naumann et al., 2018). An increase in drought-prone areas has adversely affected the productivity of agricultural crops. By 2050, water shortage is expected to cause serious plant growth problems in arable lands and affect the two-thirds population of the world (Naumann et al., 2018). This problem is being addressed on priority by changing and improving the genetic makeup of crop plants (Awan et al., 2015; Ilyas et al., 2020).

Five different types of soil microbes, namely, bacteria, actinomycetes, fungi, protozoa, and nematodes, play an important role in increasing plant and soil health (Ali et al., 2019; Msimbira and Smith, 2020). Microbial presence in plant soil depends on the soil’s temperature, pH, availability of water, and nutrients. A symbiotic relationship exists between plants and beneficial soil microorganisms wherein the microbes help the plants in nitrogen acquisition, water uptake, and survival during stress (Msimbira and Smith, 2020; Xiong et al., 2021). According to estimates, rhizobia contribute to 50% of the biological nitrogen fixation on earth (Msimbira and Smith, 2020). Various functions performed by beneficial soil microorganisms include accumulation and cycling of organic compounds, stimulation of nutrient mineralization, and production of plant growth hormones. Plants release carbon in their root systems by rhizodeposition in the form of root exudates that sustain the soil microbiome in plant roots (Khan et al., 2020). Studies have reported that 5–21% of the carbon fixed during photosynthesis is released into the rhizosphere, which can be defined as the area of soil under the biochemical influence of plant roots (Hartman and Tringe, 2019; Gontia-Mishra et al., 2020), and constitutes an important nutrient source for soil microbial community (Xiong et al., 2021).

Plants growing in the soil develop a close relationship with soil microbes residing around, on, or inside the plant roots. Certain soil microbes, including bacteria, archaea, fungi, and oomycetes, colonize the root surface and inner root tissues (Gouda et al., 2018), thus playing an important role in inducing drought stress tolerance in host plants (Hartman and Tringe, 2019). The selection of microbes with greater resistance could be useful in developing abiotic resistance in important crop plants. A few bacterial and fungal species that provide a better response during stress conditions have already been identified. Although no definitive spatial boundary has been defined for the rhizosphere, it is estimated to extend approximately 1–5 mm from the root surface to the surrounding soil (Hartman and Tringe, 2019). Rhizospheric microbiomes contain abundant bacterial and fungal communities that play a key role in relation to soil and plants (Danish et al., 2020; Lin et al., 2020). Common inhabitants of the rhizosphere include beneficial plant-growth-promoting microorganisms, root pathogens, and root-feeding insects (Barnawal et al., 2017; Lin et al., 2020). Diversity in the rhizosphere creates ecological niches and micro-environments for different microbial species to perform beneficial interactions (Saleem et al., 2018). Other functions of beneficial rhizosphere microbes include organic matter decomposition, nitrogen fixation, phosphorus solubilization, transportation, and biocontrol of root pathogens (Danish et al., 2020; Gontia-Mishra et al., 2020).

This review aims to understand the effects of drought stress on the morphological, physiological, and molecular traits of plants. Moreover, we discuss how soil microbial communities are useful in minimizing or reducing the effects of drought stress in various plants. In this review, we explore the recent progress achieved by researchers in understanding the interaction between plant growth-promoting rhizobacteria (PGPR) and crop plants under drought stress conditions. We also explore several useful aspects of PGPR and crop plants, such as developmental stages, genotypes, and climatic variables, which have not been covered in detail earlier. We conclude the review with a discussion on technical challenges and limitations in recent research methods with regard to drought stress and soil microbe interactions along with future directions and suggestions.

### Effects of Drought Stress on Plant Life

**FIGURE 1:**
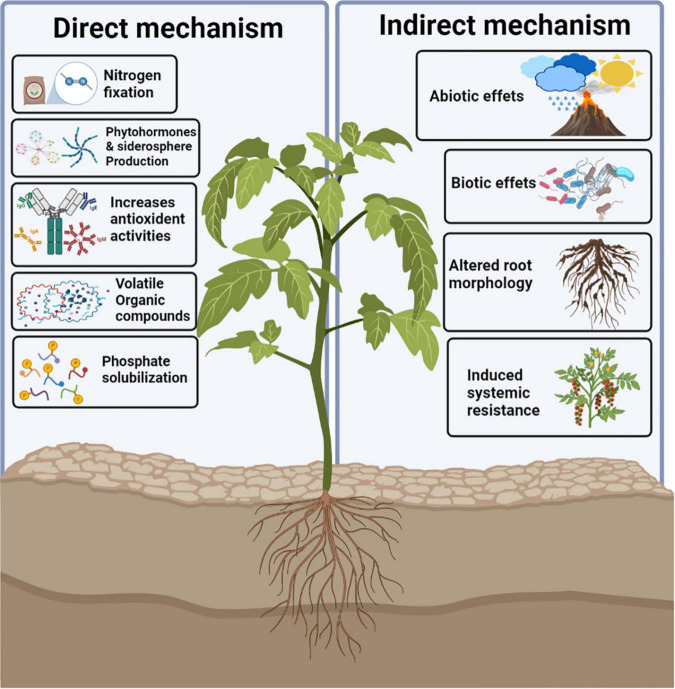
Morphological, physiological, and molecular response to drought stress in plants.

Impaired germination along with poor stand establishment is the basic and foremost effects of dehydration stress on plants (Javed et al., 2016; Lin et al., 2020). It has been reported that inadequate availability of irrigation water causes closure of stomata, reduced production of biomass, and stunted growth and development in crop plants (Ilyas et al., 2020; Marchin et al., 2020). In response to drought stress, plants reduce the root, shoot, and leaf growth, as well as water uptake, leaf water potential, transpiration rate, and turgor presser, leading to decreased relative water content (RWC) and cell turgor, along with damage to the plant cell (Ali et al., 2016; Javed et al., 2016). Different morphological, physiological, and transcriptional responses to drought stress on plants are shown in Figure 1. Researchers observed a negative impact of water stress on plant height and leaf area index in wheat and maize (Javed et al., 2016; Ilyas et al., 2020). Drought stress increases the temperature of the plant owing to dehydration in the cells (Ilyas et al., 2020) and also causes injury by interrupting the water balance of the plant body. However, the adverse effect of osmotic stress depends on its severity and duration, as well as on the growth stage of an individual crop. Moreover, drought stress has different impacts on the plant roots and leaves; root growth is favored over leaf growth in such conditions owing to rapid osmotic adjustment, which allows partial turgor recovery and reestablishment of osmotic gradients for water uptake (Marchin et al., 2020; Zhang M. et al., 2020). Any further decrease in the loosening ability of the cell wall allows the roots to resume their growth under drought conditions. Drought stress reduces the RWC, transpiration rate, and leaf water potential in plants while increasing the leaf temperature (Ferreira et al., 2019). Exposure of wheat plants to drought stress resulted in reduced plant height, a number of tillers, flag leaf area, and biological yield (Ahmed et al., 2011; Javed et al., 2016). Reduced plant germination was reported under dehydration stress in maize and sorghum (Ferreira et al., 2019; Ilyas et al., 2020). In contrast, leaves exhibited less osmotic adjustment under similar stress conditions and maintained their wall loosening ability, which led to growth inhibition (Javed et al., 2016; Ilyas et al., 2020). Water use efficiency is also an important feature that determines the limited water stress in plants and can be enhanced by improving agriculture practices that encourage curtailed water evaporation (Hatfield and Dold, 2019). Improved water use efficiency under drought has been reported in wheat (Javed et al., 2016), maize (Ilyas et al., 2020; Lin et al., 2020), and sorghum (Ferreira et al., 2019).

Although drought stress does not affect photochemical activities at the initial stage, it reduces the maximum quantum yield from photosystem II at an advanced stage; however, the yield can be completely recovered after 3 days of re-watering (Ferreira et al., 2019; Ilyas et al., 2020). Variation in photosynthesis rates under drought stress has been observed in several crops (Pinheiro and Chaves, 2011; Blum, 2017). Photosynthetic inhibition and downregulation during osmotic stress interact with the production, growth, and survival of crop plants (Ferreira et al., 2019; Khan N. et al., 2019). A strong association has been reported between stomatal conductance and photosynthetic rate in plants subjected to drought stress (Blum, 2017; Bo et al., 2017).

Long prevailing drought reduces stomatal conductance, stem conductivity, and carbon dioxide (CO_2_) assimilation leading to reduced ribulose biphosphate activity. This is because metabolic impairment decreases the photosynthetic rate in plants, ultimately resulting in the reduction of ribulose biphosphate synthesis. Water stress restricts the photosynthetic assimilation of CO_2_ because of closed stomata and restricted diffusion of CO_2_ under water stress; second, it inhibits the metabolism of CO_2_ (Blum, 2017; Marchin et al., 2020). It has been further reported that the reduced CO_2_ assimilation under drought conditions is caused not by increased CO_2_ concentration in the environment (Marchin et al., 2020) but by the closure of stomata that minimizes water loss by reducing internal CO_2_ levels (Awan et al., 2015). It has been observed that drought-stressed plants disrupt the carbohydrate flow and increase the accumulation of epicuticular waxes and soluble sugars in stressed leaves (Pour-Aboughadareh et al., 2017). Drought stress reduces sucrose and starch contents in wheat grains (Lu et al., 2019). The application of drought stress influenced the accumulation of amylopectin, amylose, sucrose, and total starch contents (Lu et al., 2019).

### Plant Growth-Promoting Rhizobacteria and Their Formulations

Species, such as *Pseudomonas fluorescens*, *Pseudomonas putida*, *Pseudomonas aeruginosa*, *Bacillus subtilis*, and other *Bacillus* sp., are widely used for the commercial production of PGPR. Various fermentation technologies have been used to formulate potential PGPR isolates using organic and inorganic carriers. Ideal formulations should possess characteristics, such as long shelf life, satisfactory water solvency, tolerance to adverse environmental conditions, compatibility with other agrochemicals, and non-phototoxicity. Research has proved that mixed strain formulations yield better results than individual strains because mixed strain formulations can help in combating multiple stresses and diseases in addition to promoting plant growth and development. In addition to the formulation, the method used for delivering the PGPR to the plants is also important to achieve the desired results. Usual delivery methods include bio-priming, seed treatment, foliar application, foliar spray, fruit spray, soil application, and seeding dip.

### Role of Plant Growth-Promoting Rhizobacteria in Growth and Development of Plants Under Drought Stress

The role of PGPR in nutrient management, biocontrol activity, plant growth, and development is well established (Gouda et al., 2018; Fabiańska et al., 2019). These rhizosphere-inhabiting microbes help the plants in their growth and development through diverse mechanisms (Gouda et al., 2018). Currently, research on their role in tolerating biotic and abiotic stresses is gaining importance (Meenakshi et al., 2019; Woo et al., 2020). Osmotic stress strongly affects plant growth, development, and soil microbial activity (Gowtham et al., 2020). Various pathways involved in rhizosphere microbe-mediated osmotic stress tolerance in crop species have been studied (Gouda et al., 2018; Fabiańska et al., 2019). These mechanisms include alteration in root architecture, phytohormonal activities, osmolyte accumulation, antioxidant defense, and transcriptional response to defense (Hartman and Tringe, 2019). Soil microbes have been intensively incorporated in agriculture production systems owing to their potential to promote plant growth, abiotic stress resistance, and management of plant diseases (Goswami and Deka, 2020). These microbes play a vital role in plant growth through the production of bacterial phytohormones, exopolysaccharides (EPSs), and associated metabolites by increasing the nutrient availability in the rhizosphere and protecting the plants from abiotic stresses (Naseem et al., 2018; Goswami and Deka, 2020). However, the reaction of bacteria to drought stress varies depending on stress duration, intensity, growth stage, and plant species (Naseem et al., 2018). Water stress directly affects the soil processes in several ways, including stressing the microorganisms (Goswami and Deka, 2020). Under drought conditions, soil microbes adjust their osmotic conditions and try to maintain their hydration by accumulating solutes for retaining water in their cells (Shirinbayan et al., 2019). An indirect effect of drought stress on soil processes is the alteration in the supply of substrates to the rhizosphere bacteria through dissolution, diffusion, and transport (Shirinbayan et al., 2019). Plant growth-promoting bacteria are involved in accelerating flowering, early senescence, and seed set stages (Gowtham et al., 2020), and the early flowering strategy is associated with the drought escape mechanism (Meenakshi et al., 2019). Diazotrophic bacteria are linked with agave roots under drought stress and can enhance plant growth under drought conditions (Zarei et al., 2019; Abbasi et al., 2020). Similarly, the role of bacteria in plant growth under limited water conditions has been demonstrated in previous studies (Meenakshi et al., 2019); for example, bacterial inoculation improved the water use efficiency, root and shoot biomass, RWC, and membrane stability index, thereby reducing the adverse effect of drought stress in wheat and tomato plants (Meenakshi et al., 2019; Abbasi et al., 2020). *P. fluorescens* DR7 enhanced plant growth under drought stress conditions by increasing the soil moisture in foxtail millet (Niu et al., 2018). Enhanced plant growth after inoculation with plant growth promoter regulators, that is, *P. putida*, *Azospirillum lipoferum*, *P. fluorescens* P1, and *P. fluorescens* P8 has been reported in maize when drought-subjected plants were compared with non-treated ones (Sandhya et al., 2010; Khan and Bano, 2019; Zarei et al., 2019). Research has confirmed that endophytic bacterial strains MKA2, MKA3, and MKA4 mitigate drought stress in wheat plants (Meenakshi et al., 2019). Application of plant growth-promoting bacterial strain *B. subtilis* SF48 enhanced growth and RWC in tomato plants under drought stress conditions compared with that in control plants (Gowtham et al., 2020; Table 1).

**TABLE 1:** Alteration in root morphology, plant growth, and development by PGPR under drought stress.

Soil microbe/Strain	Plant species	Effect under drought	References
*Azospirillum brasilense*	Tomato	Enhanced lateral root and root hair development	Molina-Favero et al., 2008
*Azospirillum brasilense* Az39	Rice	Improved root growth and mitigated osmotic stress	Cassán et al., 2009
*Paenibacillus polymyxa*	Wheat	Enhance plant survival and biomass production under osmotic stress	Arzanesh et al., 2011
*Azospirillum brasilense* Sp245	Wheat	Increased growth and expansion of xylem in the coleoptile of inoculated plant for easy conduction of water	Timmusk et al., 2014
*Paenibacillus polymyxa* B2	Arabidopsis	Induction of early response to dehydration stress	Timmusk et al., 2014
*B. thuringiensis* NEB17	Soybean	Modification of root structure, root length, root ABA	Prudent et al., 2015
*Bacillus megaterium* BOFC15	Arabidopsis	Alter root architecture system	Zhou et al., 2016
*P. putida* FBKV2	Maize	Encouraged root and shoot growth, dried biomass weight and reduced stomatal conductance in the plant	Vurukonda et al., 2016
*Azospirillum brasilense* SP-7	Maize	Higher drought tolerance, higher biomass production and chlorophyll contents	Curá et al., 2017
*H. seropedicae* Z-152	Maize	Higher drought tolerance, higher biomass production and chlorophyll contents	Curá et al., 2017
*Azospirillum sp.* Az19	Maize	Improve the growth and productivity of the plant under water stress	García et al., 2017
*O. pseudogrignonense* RJ12, *Pseudomonas sp.* RJ15, *B. subtilis* RJ46	Mungbean	Increase root length, shoot length, plant dry weight and root recovery intension	Saikia et al., 2018a
*B. subtilis*	Maize, Common bean	Improved water use efficiency and growth	de Lima et al., 2019
*M. luteus* 3.13 and 4.43	Sunflower	Enhanced the weight, area, volume, length, diameter, and surface	Namwongsa et al., 2019
*V. paradoxus* RAA3 *O. anthropic* DPC9 *P. palleroniana* DPB13 *P. fluorescens* DPB15 *P. palleroniana* DPB16	Millet	Improve the growth and nutrient concentrations in plant leaves under drought conditions	Chandra et al., 2019
*B. subtilis* GOT9	Arabidopsis, Canola	Drought stress tolerance, growth, and development of lateral roots	Woo et al., 2020
*Pseudomonas lini* and *Serratia Bizio plymuthica*	Jujube	Improve plant height, RWC, root, and shoot dry weight	Zhang Y. et al., 2020
*Bacillus licheniformis* FMCH001	Maize	Improved water use efficiency and increased root dry weight	Akhtar et al., 2020

### Mechanisms Employed by Plant Growth-Promoting Rhizobacteria for Drought Stress Tolerance

**FIGURE 2:**
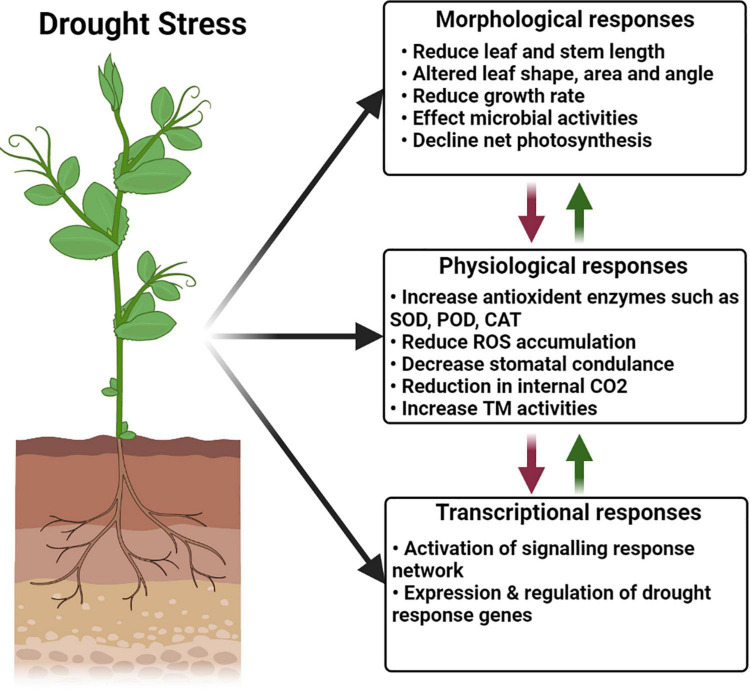
Various mechanisms are adopted by PGPR to eliminate drought stress.

With the help of root-associated bacterial communities, plants adopt various mechanisms to tolerate drought stress. There are two main mechanisms adopted by PGPR to overcome osmotic stress in plants: direct and indirect. Direct mechanisms are phenomena occurring inside the plant and affect the plant metabolism directly, whereas indirect mechanisms occur outside the plants (Vurukonda et al., 2016). The major mechanisms adopted by PGPR to overcome drought stress include alteration in root morphology and production of osmolytes, antioxidants, phytohormones, extracellular polymeric substance (EPS), and volatile organic compounds (VOCs), siderophores, and 1-aminocyclopropane-1-carboxylate (ACC) deaminase. The various mechanisms are presented in detail in Figure 2. These mechanisms may be direct or indirect depending upon the host plant, as well as the biotic and abiotic stress factors (Gouda et al., 2018).

### Change in Structure and Morphology of Plant Root System

The term root morphology/architecture encompasses the root depth, root angle, density, root volume, and biomass (Saleem et al., 2018). Plants dynamically modify their root morphology to manage drought stress. Water stress is directly correlated with root morphology because a long and more extensive root architecture allow the plants to uptake more amount of water from the soil (Saleem et al., 2018; Mishra et al., 2020). Drought-tolerant plants tend to have greater rooting depth, density, root volume, and weight (Jochum et al., 2019). Although plants prefer root growth overshoot growth under drought stress conditions, even that is hindered under severe stress (Vurukonda et al., 2016; Barnawal et al., 2017). Variations in root morphology under limited water conditions are species-specific (Mishra et al., 2020).

Root-associated microorganisms play an important role in maintaining the health of the host plant. However, the existence of these microorganisms depends on soil chemistry, perturbations in the surrounding abiotic environment, as well as plant genotype and phenotype. Further, it has been noted that the composition of soil microorganisms varies at different rooting depths because soils exhibit specific patterns of bacterial communities at specific depths (Zhang et al., 2018); furthermore, rhizospheres from root sections obtained at different depths have distinct microbiota (Jochum et al., 2019; Gontia-Mishra et al., 2020). The plant root system affects the health, fitness, and productivity of plants by changing the root length, surface area, density, volume, and biomass. Rhizospherical microbial communities influence these phenotypic traits by altering the processes occurring in the soil (Goswami and Deka, 2020; Lin et al., 2020). Hence, enhanced root development improves nutrient uptake from the soil and the water absorption capacity of plants (Lin et al., 2020). During water stress, bacteria change the elasticity of the root cell membrane, which is the foremost step in enhancing drought tolerance (Dimkpa et al., 2009; Lin et al., 2020). Altered root metabolites play an important role in the selection of certain species (Mahdi Dar et al., 2018; Xu et al., 2018). A positive correlation has been observed between increased carbohydrates in roots and carbohydrate transporters in *Actinobacteria* (Xu et al., 2018). During drought stress, the rhizosphere microbial community increases the root surface area and fine root production on one hand and reduces stress-associated volatile emissions on the other, leading to a marked improvement in plant performance (Saleem et al., 2018). Inoculation of maize plants with *P. putida* improved the leaf water potential, RWC, and plant biomass when exposed to drought stress (Sandhya et al., 2010). Bacterial inoculation in wheat plants improved the formation of lateral roots and enhanced root growth, thereby increasing the water uptake under drought conditions (Mahdi Dar et al., 2018). Inoculation with *Bacillus thuringiensis* and *Azospirillum brasilense* improved the specific root area and length along with the root projection area in common beans (Armada et al., 2014; Mahdi Dar et al., 2018). Inoculation with *Ochrobactrum* spp. strain NBRISH6 improved the root length, dry weight, and hairs in maize under water stress regimes (Mishra et al., 2020).

### Production of Osmolytes

Plants initiate metabolic changes for survival during drought stress, leading to the accumulation of compatible osmolytes, such as proline, glycine betaine (GB), polyamines, sugars (trehalose, polyols), polyhydric alcohols, and dehydrins. Plant growth-promoting microorganisms (PGPMs) introduce osmotic stress by increasing the accumulation of osmolytes in the host plant (Gontia-Mishra et al., 2020). Recent research reported that *Azospirillum* spp. is responsible for the accumulation of such compatible solutes under limited water conditions (García et al., 2017).

Proline content is directly linked with drought stress, and it increases proportionately with the severity of the stress (Ortiz et al., 2015; Abdela et al., 2020). High proline content is involved in cell membrane protection and maintenance of cell water status during limited water supply (Ortiz et al., 2015). Therefore, assessing proline content is important for evaluating drought stress tolerance and sensitivity in crop plants (Abdela et al., 2020). Application of *P. putida* strain GAP-P45 improved the accumulation of proline in maize plants subjected to drought stress (Sandhya et al., 2010). Inoculating *B. thuringiensis* in maize plants under water stress increased their shoot proline content when compared with that in control (Armada et al., 2014). The application of *Paenibacillus polymyxa* on tomato cultivars caused higher proline secretion to overcome the drought stress (Ghosh et al., 2019). Inoculation with *Streptomyces* spp. and *Mesorhizobium ciceri* spp. increased the proline contents in tomatoes (Abbasi et al., 2020) and chickpeas (Abdela et al., 2020), respectively.

Upregulation of GB content under drought stress may be attributed to certain key enzymes of gene expression (Zhang et al., 2010). Enhanced accumulation of GB content, which is a major cause of reduced water loss, was reported in plants subjected to PGPR inoculation under drought conditions (Nadeem et al., 2010; Bashan et al., 2014). Similarly, drought-stressed plants inoculated with *B. subtilis* and *Pseudomonas* spp. exhibited higher GB content than non-treated plants (Sandhya et al., 2010). Endogenous accumulation of proline and GB has been observed in mung beans when plants were inoculated with *P*. *aeruginosa* (Sarma and Saikia, 2014). Trehalose is an important signaling molecule in plants and plays an important role in drought stress tolerance. As a non-reducing disaccharide, this osmoprotectant stabilizes the cell membrane by modulating the antioxidant enzyme activity (Barnawal et al., 2017). The application of even a minute amount of trehalose to maize roots is sufficient to generate the stress tolerance signal pathway. Inoculation with *A. brasilense* in maize plants upregulated the trehalose-producing genes, leading to enhanced drought tolerance and biomass production (Rodríguez-Salazar et al., 2009; Curá et al., 2017).

Choline is also an important osmolyte that plays a role in overcoming the drought stress by accumulating GB, thereby enhancing the dry matter and leaf water contents. Further, increased choline contents in maize and wheat enhanced the nutritional value of food additives (Zhang et al., 2010; Iqbal, 2018). Various studies have demonstrated the evident role of soil microbial communities in the accumulation of choline as a precursor of GB metabolism (Rocha et al., 2019). Polyamines are another type of osmolytes associated with root growth under drought stress. The introduction of *A. brasilense* strain A39 helped rice plants accumulate polyamines in the seedlings under osmotic stress conditions (Cassán et al., 2009). Another research reported that inoculation of cowpea plants with *Rhizophagus irregularis* enhanced both chlorophyll and carotenoid contents under severe water stress (Rocha et al., 2019; Table 2).

**TABLE 2:** Photosynthetic pigments and osmolytes produced by PGPR to mitigate drought stress.

Soil microbe/Strain	Plant species	Effect under drought	References
*Azospirillum brasilense*	Maize	Trehalose translocated to the maize roots and triggered stress tolerance pathways in the plants	Rodríguez-Salazar et al., 2009
*A. lipoferum*	Maize	Increase gibberellins synthesis and alleviate drought stress	Cohen et al., 2009
*P. putida* GAP-P45	Maize	Accumulation of proline improved plant biomass, relative water content, and leaf water potential	Sandhya et al., 2010
*B. subtilis* GB03	Arabidopsis	Enhance metabolic level of choline and gerbilline, improve leaf RWC under drought stress	Zhang et al., 2010
*P. aeruginosa* JHA6	Pepper	Increased biomass production as well as chlorophyll content of inoculated plants and nutrient uptake	Gupta et al., 2019
*Bacillus amyloliquefaciens* ROH14	Pepper	Increased biomass production as well as chlorophyll content of inoculated plants and nutrient uptake	Gupta et al., 2019
*P. putida* H-2–3	Soybean	Secretion of gibberellins and improved plant growth	Kang et al., 2014
*P. aeruginosa* GGRJ21	Mungbean	Accumulation of proline and GB under drought stress	Sarma and Saikia, 2014
*P. putida* *B. thuringiensis*	White clover	Decreased stomatal conductivity, electrolyte leakage, and proline content	Ortiz et al., 2015
*B. aquimaris S 4.43*	Sunflower	Improved the chlorophyll level and photosynthesis rate under drought	Namwongsa et al., 2019
*Rhizophagus irregularis*	Cowpea	Enhanced chlorophyll and carotenoid contents under drought stress	Rocha et al., 2019
*B. velezensis* 5113	Wheat	Higher chlorophyll contents, plants survival under drought stress	Abd El-Daim et al., 2019
*P. chinense* (P1), *B. cereus* (P2), *P. fluorescens* (P3)	Wheat	Enhanced production of proline, antioxidant enzymes, and lipid peroxidation	Khan N. et al., 2019
*Pseudomonas sp.* N66	Sorghum	Production of proline, glutamic acid, and choline	Carlson et al., 2020
*A. xylosoxidans*	Maize	Enhances photosynthetic rate, stomatal conductance, chlorophyll a, total chlorophyll, and carotenoids contents	Danish et al., 2020
*Mesorhizobium ciceri* CP41 *P. fluorescens* G.	Chickpea	Improved relative water content, proline, total soluble sugar, total chlorophyll, and carotenoid contents	Abdela et al., 2020

### Extracellular Polymeric Substance Production

Extracellular polymeric substances (EPSs) are high-molecular-weight, biodegradable polymers composed of monosaccharide residues and their derivatives and are biosynthesized by a wide range of bacteria, algae, and plants (Sanalibaba and Cakmak, 2016). EPSs play a central role in maintaining water potential, aggregating soil particles, ensuring obligate contact between plant roots and rhizobacteria, and sustaining the host under stress or pathogenic conditions, thus bearing direct responsibility for plant growth and crop production (Naseem et al., 2018). EPSs play an important role in protecting land plants from drought stress by maintaining the plant-microbes interaction (Khan and Bano, 2019) and are extremely useful in various industries, owing to their bioremediation, stabilizing, thickening, coagulating, gel-developing, suspending, and film-forming properties. PGPR could be effectively used to overcome the drastic effects of water stress by increasing the production of EPSs and forming rhizosheaths around the roots, protecting them from dehydration. Application of EPS-producing PGPR can prove helpful in mitigating water deficiency and consequently increasing global food security (Khan and Bano, 2019); however, the outcome of PGPR application to osmotic stress depends not only on the stress intensity and duration but also on the plant species and its growth phase (Table 3).

**TABLE 3:** Mitigation of drought stress through EPSs produced by PGPR.

Soil microbe/Strain	Plant species	Effect under drought	References
*P. putida* GAP-P45	Sunflower	Alleviation of drought stress and exopolysaccharide production	Sandhya et al., 2009
*P. putida* GAP-P45	Maize	Improve water holding capacity and exopolysaccharide production	Sandhya and Ali, 2015
*Proteus penneri* (Pp1), *P. aeruginosa* (Pa2), *Alcaligenes faecalis* (AF3)	Maize	Improve EPS production, leaf area, and plant biomass	Naseem et al., 2018
*Bacillus amyloliquefaciens* HYD-B17, *Bacillus licheniformis* HYTAPB18, *B. subtilis* RMPB44	Arabidopsis	Enhanced EPS production under drought	Vardharajula and Ali Sk, 2014
*Bacillus amyloliquefaciens* FZB42	Arabidopsis	Exopolysaccharide production and induce systemic drought tolerance	Lu et al., 2018
*B. methylotrophicus* 5.18	Sunflower	Enhanced EPS production along with other drought tolerance traits	Namwongsa et al., 2019
*P. chinense* P1 *B. cereus* P2	Wheat	Improved production of EPS, enhanced plant growth, and drought tolerance	Khan and Bano, 2019
*P. aeruginosa* ZNP1 *B. endophyticus* J13	Arabidopsis	Exhibited increased EPS production under osmotic stress	Ghosh et al., 2019

Production of EPSs by PGPR significantly affects the plant growth, development, and drought tolerance capacity (Subramaniam et al., 2020) as these microbes can survive under low-moisture soils through nodule formation. EPSs can provide a micro-environment that dries very frequently in comparison with the surrounding soil but stays hydrated by holding water and thus protecting the bacteria and plant roots against desiccation (Subramaniam et al., 2020). EPS production by bacteria enhanced and improved the ability of soil in balancing the water potential and sustaining soil aggregation, thereby improving the nutrient uptake and resulting in the enhanced growth and development of the plants and protection from dehydration (Subramaniam et al., 2020). The EPS produced by PGPR, such as *Rhizobium leguminosarum*, *Agrobacterium vinelandii*, *Bacillus drentensis*, *Enterobacter cloacae*, *Agrobacterium* spp., *Xanthomonas* sp., and *Rhizobium* sp., are vital for nourishing the soil and maintaining crop production (Mahmood et al., 2016). The role of PGPR in enhancing desiccation tolerance in plants through EPS production was observed in *Arabidopsis* (Ghosh et al., 2019), maize (Khan and Bano, 2019), and sunflower (Sandhya et al., 2009). Inoculation with EPS-producing *R. leguminosarum* LR-30, *M. ciceri* CR-30, and CR-39, and *Phaseolus phaseoli* MR-2 demonstrated their mutual interactions with wheat during drought conditions. Bacterial strains *Proteus penneri* Pp1, *P. aeruginosa* Pa2, and *Alcaligenes faecalis* AF3 can produce EPS and maintain soil moisture, contents, biomass, root and shoot length, and leaf area of the plants (Naseem et al., 2018).

### 1-Aminocyclopropane-1-Carboxylate Deaminase Activity

Plant growth-promoting microorganism can synthesize ACC in plants under drought stress (Chandra et al., 2019; Danish et al., 2020). On exposure to drought stress, the plant hormone ethylene endogenously regulates plant homeostasis and restrains root and shoot growth along with leaf expansion, ultimately restricting the plant growth (Li et al., 2017). ACC is an immediate precursor of ethylene (Danish et al., 2020; Gowtham et al., 2020); the bacterial ACC deaminase enzyme converts the ACC to ammonia and α ketobutyrate and inhibits ethylene production in plants (Danish and Zafar-ul-Hye, 2019; Jochum et al., 2019). High ACC deaminase activity of *Pseudomonas* strains under drought stress has been observed in millet (Niu et al., 2018; Chandra et al., 2019). Recent studies suggested that under drought stress, inoculation with ACC deaminase-producing rhizobacteria can improve the negative effects of reactive oxygen species (ROS), which is beneficial for plant survival (Chandra et al., 2019; Danish et al., 2020). Oxidative stress on tomato and pepper plants was alleviated by ACC deaminase, and their fresh and dry weight increased when compared with that of the plants of the control treatment (Gupta and Pandey, 2019; Gowtham et al., 2020). The effect of ACC deaminase-producing rhizobacteria under drought stress conditions has been reported in wheat (Chandra et al., 2019), maize (Danish et al., 2020), millet (Chandra et al., 2019), rice (Zhang Y. et al., 2020), mint (Asghari et al., 2020), and tomato (Gowtham et al., 2020). ACC deaminase-producing bacteria *B. Subtilis* Rhizo SF 48 protects tomato plants against drought-induced oxidative damage (Gowtham et al., 2020). Improvement in maize growth and yield under drought conditions was observed because of the accumulation of ACC deaminase by *E. cloacae* and *A. xylosoxidans* (Danish et al., 2020). The adverse effect of drought stress on growth and productivity was eliminated by ACC deaminase-producing bacteria in pea plants (Arshad et al., 2008). Similarly, inoculation with ACC deaminase-producing *Achromobacter piechaudii* ARV8 in tomato and pepper significantly reduced the production of ethylene under drought stress (Mayak et al., 2004; Gowtham et al., 2020).

Improved water uptake efficiency and longer root growth under drought stress have been achieved by inoculation with ACC deaminase-producing *P. fluorescens* in pea plants (Zahir et al., 2008). Axenic studies demonstrated that inoculation with ACC deaminase-producing rhizobacteria increased root–shoot length, root–shoot mass, and the lateral number of roots of wheat plants compared with that of the control. Better development of roots helped the plants acquire water and nutrients resulting in improved growth and yield under drought stress (Ilyas et al., 2020). Co-inoculation with ACC deaminase-producing *Bacillus* isolate 23-B and *Pseudomonas* 6-P in conjunction with *M. ciceri* for mitigation of drought stress and plant growth promotion under drought conditions in chickpea significantly improved germination, root and shoot length, and the fresh weight of plants. Among the treatments, co-inoculating 23-B with *M. ciceri* was efficient under drought stress (Palika et al., 2013). Similarly, inoculation with ACC deaminase-producing *Bacillus licheniformis* K11 alleviated drought stress in pepper (Lim and Kim, 2013; Table 4).

**TABLE 4:** Improved ACC deaminase activity and reduced ethylene production by PGPR under drought stress.

Soil microbe/Strain	Plant species	Effect under drought	References
*A. piechaudii*	Tomato	Reduce the Ethylene production	Mayak et al., 2004
*B. thuringiensis* AZP2	Wheat	High Phosphate solubilizing efficiency, ACC deaminase activity, improved crop growth and biomass	Timmusk et al., 2014
*Paenibacillus polymyxa* B	Wheat	High Phosphate solubilizing efficiency, ACC deaminase activity, improved crop growth and biomass	Timmusk et al., 2014
*P. fluorescens* DR7	Millet	Promote plants growth under drought stress, ACC deaminase activity	Niu et al., 2018
*B. Brevibacterium S91*	Tea	Improved ACC deaminase activities and IAA production	Borah et al., 2019
*Pseudomonas* sp. N66	Sorghum	Lower the ethylene level by improving the ACC deaminase activities	Carlson et al., 2020
*E. aerogenes*	Rice	Improves rhizosphere health under mild drought stress through ACC deaminase activity	Zhang Y. et al., 2020

### Production of Phytohormones

Phytohormones are small, endogenous, lower molecular-weight molecules responsible for activating an effective defense response against biotic and abiotic stresses. A group of ten interconnected phytohormones, such as abscisic acid (ABA), indole 3 acetic acid (IAA), auxin, cytokinin (CK), gibberellin (GA), ethylene (ET), salicylic acid (SA), strigolactones (STs), jasmonate (JA), and brassinosteroid (BRs), help plants in their defense mechanism (Raheem et al., 2018). Among these plant hormones, ABA, JA, SA, and ET are considered abiotic stress response hormones (Raheem et al., 2018). Phytoproducts excreted from plant roots control the soil microbial community by altering the rhizospheric soil chemistry (Jochum et al., 2019). Possible reasons for the rhizobacteria-mediated plant drought tolerance include, (1) development of phytohormones, such as ABA, GA, CK, and IAAs; (2) reduced ethylene levels in roots because of ACC deaminase; (3) mediated systemic tolerance by bacterial compounds; and (4) bacterial EPSs (Vurukonda et al., 2016; Table 5).

**TABLE 5:** Improvement in phytohormone production by PGPR under drought stress.

Soil microbe/Strain	Plant species	Effect under drought	References
*P. brassicacearum* STM196	Arabidopsis	Enhanced ABA decreased leaf transpiration	Bresson et al., 2013
*G. diazotrophicus* PAL5	Sugarcane	Inoculation activated the ABA-dependent signaling genes conferring drought resistance	Vargas et al., 2014
*Azospirillum sp.*	Wheat	IAA enhanced root growth, lateral roots formation, and increased uptake of water and nutrients	Hosseini et al., 2017
*Bacillus amyloliquefaciens* S-134	Wheat	Higher IAA production under water stress	Raheem et al., 2018
*M. luteus* S4.43	Sunflower	Enhanced the IAA production under drought	Namwongsa et al., 2019
*H. huttiense* RCA24	Rice	IAA producer under drought stress	Andreozzi et al., 2019
*B. endophyticus* J13 *B. tequilensis* J12	Tomato	Stress-induced increase in the levels of phytohormones, gibberellic acid, auxin, and cytokinin	Ghosh et al., 2019
*B. subtilis* DHK	Maize	Production of IAA and stimulates the transcription of ACC synthase enzyme	Sood et al., 2020

In addition to the production of phytohormones, such as IAA, GA, CK, and ethylene, the solubilization of phosphates, nitrogen fixation, and generation of siderophores are all direct mechanisms of drought effect mitigation (Gontia-Mishra et al., 2020; Gowtham et al., 2020), which stimulates root proliferation, increasing the absorption of nutrients, and thus promoting the plant growth (Raheem et al., 2018). Phytochromes, such as IAA, GA, ethylene, ABA, and CK, produced by plants are essential for their growth and development (Andreozzi et al., 2019; Borah et al., 2019). Phytohormones help plants avoid or survive abiotic stress in stressful environments (Andreozzi et al., 2019; Borah et al., 2019). In addition, PGPR can synthesize phytohormones that promote the growth and division of plant cells that are resistant to abiotic stresses (Ghosh et al., 2019).

Indole 3 acetic acid is an auxin that is physiologically involved in plant growth and development. Increased root growth and formation of lateral and root hairs for higher water and nutrient uptake were reported in various plant species after inoculation with IAA to manage drought stress (Dimkpa et al., 2009; Vandana et al., 2020). IAA increases plant resistance to drought stress because it produces *Azospirillum* (Dimkpa et al., 2009). Bacterial hormone production and their ability to stimulate endogenous hormones play an important role in enhancing drought tolerance (Ghosh et al., 2019). In tomato plants, *A. brasilense* produces nitric oxide gas, which functions as a signaling molecule in the IAA-inducing pathway and helps in the development of adventitious roots (Creus et al., 2005; Molina-Favero et al., 2008). Maize seedlings inoculated with *A. brasilense* increased their relative and absolute water quality in comparison with non-inoculated plants under drought stress (Danish et al., 2020). Although microbial treatment in plants lowered their water potential, it enhanced the root production, biomass, foliar area, and leaf and root proline accumulation (Vurukonda et al., 2016). Inoculation with *A. brasilense* Sp245 in wheat under drought conditions resulted in high grain yield and mineral quality (Mg, K, and Ca), with improved relative and absolute water content, water capacity, and apoplastic water fraction and lower volumetric cell wall elasticity, suggesting that “elastic change” is crucial during increased drought status. Similarly, *Azospirillum* introduced to wheat induced a decreased water potential and increased water quality of leaves because plant hormones, such as IAA, secreted by the bacteria enhanced the general and lateral root growth by increasing the water and nutrient consumption under drought conditions (Arzanesh et al., 2011). Production of phytohormones, such as IAA, improves maize growth with the help of PGPM, including *E. cloacae* and *A. xylosoxidans* (Danish et al., 2020; Table 6).

**TABLE 6:** Improvement in phytohormone/enzyme production by PGPR under drought stress.

Soil microbe/Strain	Plant species	Effect under drought	References
*P. fluorescens*	Green gram	Production of catalase enzyme	Saravanakumar et al., 2011
*B. thuringiensis*	Maize	Improved nutrient content and water transport protein as well as reduce lipid oxidation in the stressed plant	Armada et al., 2014
*B. phytofirmans* PsJN	Wheat	Reduced oxidative stress and increased mineral components of wheat.	Naveed et al., 2014
*Bacillus megaterium* BOFC15	Arabidopsis	Scavenges ROS, Upregulates ABA biosynthesis	Zhou et al., 2016
*O. pseudogrignonense* RJ12, *Pseudomonas sp.* RJ15, *B. subtilis* RJ46	Mungbean	Elevated production of ROS scavenging enzymes and cellular osmolytes	Saikia et al., 2018b
*B. subtilis*	Maize, common bean	Decreased antioxidant activities under drought stress	de Lima et al., 2019
*Pseudomonas sp.* Strains DPB13, DPB15, and DPB16	Wheat	Improved plant growth and significantly enhanced antioxidant properties of the plants	Chandra et al., 2019
*Bacillus amyloliquefaciens* 54	Tomato	Decrease the malondialdehyde concentration and improved antioxidant activities	Wang et al., 2019
*Bacillus megaterium* STB1	Tomato	Biosynthesis of CK, auxins as well as modulation of polyamines	Nascimento et al., 2020
*Pseudomonas lini* *Serratia Bizio plymuthica*	Jujube	Decreased malondialdehyde, ABA and increased antioxidant enzyme activities	Zhang M. et al., 2020
*A. chroococcum*, *Azospirillum brasilense*	Mint	Higher ABA, proteins and soluble sugars, phenolic, flavonoid, and oxygenated monoterpenes contents	Asghari et al., 2020
*B. Subtilis* Rhizo SF 48	Tomato	Enhance plant growth, Enhance SOD, APX and ACC deaminase activity and	Gowtham et al., 2020
*Pseudomonas sp.* Strain N66	Sorghum	Augmented antioxidant capacity under drought	Carlson et al., 2020
*Bacillus licheniformis* FMCH001	Maize	Regulates the ROS level and increase CAT activities in root	Akhtar et al., 2020
*B. subtilis* DHK and B1N1	Maize	Increase antioxidant enzymatic activities and decrease reactive oxygen species	Sood et al., 2020

*Bacillus thuringiensis*-assisted *Lavandula dentata* plants grew under drought conditions because of bacteria-produced IAA, which enhanced the plant nutrition, physiology, and metabolic activity (Armada et al., 2014). Soybean plants inoculated with the gibberellin-secreting rhizobacterium *P. putida* H-2–3 demonstrated increased plant growth under drought conditions (Kang et al., 2014). ABA and GA production by *A. lipoferum* reduced the drought effect in maize plants (Cohen et al., 2009). Cellular dehydration caused ABA (a stress hormone) biosynthesis during drought stress (Kaushal and Wani, 2016). ABA is involved in water loss through regulation of the stomatal closure and the transduction tract of the following stresses. Arabidopsis plants inoculated with *A. brasilense* Sp245 had higher levels of ABA than the non-inoculated plants (Cohen et al., 2009). In *Brassica napus*, *Phyllobacterium brassicacearum* STM196 isolated from the rhizosphere increased osmotic stress in inoculated *Arabidopsis* by elevating ABA content, thereby decreasing the leaf transpiration (Bresson et al., 2013; Table 7).

**TABLE 7:** Improvement in phytohormone/enzyme production by PGPR under drought stress.

Soil microbe/Strain	Plant species	Effect under drought	References
*Bacillus megaterium* XTBG34	Arabidopsis	Production of VOC (pentyl furan) and promoting of plant growth	Zou et al., 2010
*P. fluorescens* SS101		Production of VOCs 13-tetradecadien-1-01, 2-methy-n-1-tridecene, and 2-butanone	Park et al., 2015
*B. subtilis* SYST2	Tomato	Decrease ethylene level, increase auxin, gibberellin, and cytokinin	Tahir et al., 2017
*Microbacterium sp*. EC8	Arabidopsis and tomato	Increased root and shoot biomass	Cordovez et al., 2018

### Production of Secondary Metabolites, Antioxidant Activities, and Accumulation of Reactive Oxygen Species

Secondary metabolites (SMs) are chemical compounds produced by plant cells during metabolic pathways. Major SMs include alkaloids, terpenoids, steroids, saponins, flavonoids, glycosides, phenol, and glucosinolates. Studies have been conducted previously to verify the role of plant SMs against environmental stresses that lead to enhanced production of these metabolites in plant cells through various *in vivo* and *in vitro* growth mechanisms. It has been observed that plants exposed to drought stress exhibit higher production of SMs, such as terpenes, phenols, flavonoids, and alkaloids (Badri et al., 2013). Plant metabolites and exudates, including carbohydrates, amino acids, and other nutrients, are altered in response to drought stress (Blum, 2017). Changes in the plant metabolite profile also correlate with changes in the bacterial community, with root community composition in *Arabidopsis* demonstrated to be dependent on the exudate profiles of the host plant (Badri et al., 2013). During drought, an increase in hydrolytic enzymes responsible for breaking down complex carbohydrates, such as lignin, cellulose, and other plant metabolites within the microbial communities, has been reported. Additionally, bacteria can alter ethylene production within the plant through ACC deaminase activity (Arshad et al., 2008), which in turn alters the plant growth and metabolite profiles to the benefit of plants and microbes (Mayak et al., 2004; Zhang et al., 2018). Not only the host plant can alter its exudate profile to recruit organisms but also the microbial community can influence the compounds being exuded, potentially creating a reciprocal relationship between the community and exudate profile. The extent to which the exudate profiles are a plant-driven process and the microbial community can influence that process is currently unknown.

Drought affects plant metabolism through the accumulation of ROS, including superoxide anion radicals (O^2–^), hydrogen peroxide (H_2_O_2_), hydroxyl radicals (OH), singlet oxygen (O_12_), and alkoxy radicals (RO), which can cause damage to membranes, DNA, and proteins (Vurukonda et al., 2016). These ROS also react with proteins, lipids, and DNA causing oxidative damage and impairing the normal functions of a plant cell (Vurukonda et al., 2016; García et al., 2017). Production of ROS has been demonstrated to be the key process in plant physiological response to drought, with progressive oxidative damage, stunted growth, and eventual cell death when the ROS level reaches a certain threshold (Asghari et al., 2020). ROS metabolism has been reported to be a general change across species, omics levels, and compartments in drought and exerts an impact beyond that of *Actinobacteria* (Abrahám et al., 2003; García et al., 2017). ROS metabolism and defense response transcription are correlated during drought with a variety of taxa, including *R. irregularis* and nematodes (Garcia et al., 2018). The ROS have been demonstrated to modulate the host microbiome, including the mitigation of nematode infection in soybeans and tomatoes (Prudent et al., 2015; Asghari et al., 2020). Generally, drought stress induces overproduction of ROS and destroys normal cell metabolism *via* oxidative damage of membrane proteins, DNA, and lipids (Kaushal and Wani, 2016). The MDA plays an important role in membrane lipid peroxidation. Previous studies have revealed that beneficial microbes can reduce MDA content, prevent ROS accumulation, increase antioxidant enzyme activities, and maintain plant growth under drought stress (Silambarasan et al., 2019). Inoculation of jujube with *Pseudomonas lini, Serratia Bizio plymuthica*, or their mixture significantly reduced the MDA content under drought stress (Zhang M. et al., 2020). Inoculation with the three bacterial treatments has been suggested to decrease the detrimental effects of oxidative damage caused by ROS production under stress conditions (Zhang Y. et al., 2020). Plants utilize a ROS scavenging system to remove excessive amounts of ROS to protect themselves. Host ROS metabolism genes have been reported to be associated with *Streptomyces* (a genus of *Actinobacteria*) in populus leaves, potentially demonstrating a high universal drought association between the host and its phytobiome (Garcia et al., 2018).

Superoxide dismutase and POD are the notable components that catalyze the dismutation of O^2–^ to oxygen and H_2_O_2_ (Sarker and Oba, 2018). POD plays a significant role in catalyzing hydrogen peroxide to water and oxygen (Liu et al., 2020). During environmental stress, increased ROS and MDA accumulate in plants owing to the transcription of genes, such as *PgRboHD* and *PgFE*, between the cells. Inoculation with PGPR enhanced the expression of antioxidant genes and consequently the quality of antioxidant enzyme activities (Marchin et al., 2020). The increase in enzyme activities shielded chloroplast from ROS and removed superoxides (Sarker and Oba, 2018). A study revealed that inoculated jujube seedlings exhibited notably higher superoxide dismutase (SOD) and peroxidase (POD) activities than non-inoculated seedlings and the enzyme activities increased with increased water stress (Zhang M. et al., 2020). Hence, we can conclude that treatment with the three bacteria enhanced the ability of jujube to scavenge and regulated the expression of antioxidant genes; thus, enhancing the SOD and POD activities under water stress and reducing the MDA content (Zhang Y. et al., 2020). Soil microbes enhance drought tolerance by improving the cell membrane stability through the activation of the antioxidant system (Singh et al., 2020). PGPR eliminates the oxidative damage from drought stress by manipulating the antioxidant enzymes (Singh et al., 2020). A popular plant species, basil, inoculated with a rhizobacterial consortium of *Pseudomonas* spp., *Brachypalpoides lentus*, and *A. brasilense* helped improve the chlorophyll content and antioxidant activity in plants under drought stress, resulting in the synthesis of useful substances instead of producing stress (Gowtham et al., 2020). Among the fixers of atmospheric nitrogen to plants for its nutritional needs, *Azospirillum* is a farmers’ friend that contributes to the enrichment of the soil and enables the plants to thrive under abiotic stress. A closer look at the biosynthesis of siderophores by *Gordonia rubripertincta* CWB2 suggests that the *GorA* gene under expression in *E. coli* results in the production of *GorA* hydroxylase enzyme (Esuola et al., 2016). It was observed that maize inoculated with drought tolerance-promoting species like *Pseudomonas* spp. strains, namely, *Pseudomonas entomophila*, *Pseudomonas stutzeri*, *P. putida*, *Pseudomonas syringae*, and *Prochoreutis montelli* displayed the significantly lower activity of antioxidant enzymes compared with non-inoculated plants when exposed to drought stress (Sandhya et al., 2009). *Pseudomonas* spp. DPB16 enhanced the growth of wheat plants and also modified its antioxidant properties (Chandra et al., 2019). Tomato plants inoculated with *B. subtilis* Rhizo SF 48 increased the antioxidant activities of SOD and APX enzymes (Gowtham et al., 2020). Streptomyces strains increased the MDA, H_2_O_2_, and total sugar content along with APX activity while decreasing the CAT and GPX activities under stress conditions in tomatoes (Abbasi et al., 2020).

### Accumulation of Volatile Organic Compounds

Plant growth-promoting rhizobacteria-mediated VOCs play a potential role in stimulating plant growth and induced systemic resistance (ISR) against various biotic and abiotic stresses. However, the study of the interaction between VOC with plant growth-promoting phytohormones is at a preliminary level. The earliest reported plant growth-promoting VOCs were 2,3-butanediol, acetoin, and pentyl furan (Ryu et al., 2003; Zou et al., 2010). A few VOCs described subsequently include 13-tetradecadien-1-01, 2-methy-n-1-tridecene, and 2-butanone produced by *P. fluorescens* SS101 in tobacco plants (Park et al., 2015). The VOCs formed by biocontrol strains not only help in plant growth but also prevent pathogens of bacterial and fungal nature along with nematodes while promoting resistance against phytopathogens in plants (Cordovez et al., 2018). Genera of specific bacterial species, including *Pseudomonas, Bacillus, Arthrobacter, Stenotrophomonas*, and *Serratia*, can produce VOCs that influence plant growth. Two very active VOCs, 2, 3-butanediol, and acetoin, produced by *Bacillus* spp. not only constrain fungal growth but also enhance the plant biomass (Massalha et al., 2017; Backer et al., 2018). VOCs are factors for provoking plant ISR stated that the VOCs from PGPR strains regulate disease resistance, abiotic stress tolerance, and plant growth (Tahir et al., 2017). Production of VOCs, comprising cyclohexane, 2-(benzyloxy) ethanamine, benzene, methyl, decane, 1-(*N*-phenylcarbamyl)-2-morpholinocyclohexene, dodecane, benzene (1-methylnonadecyl), 1-chlorooctadecane, tetradecane, 2,6,10-trimethyl, dotriacontane, and 11-decyldocosane, has been reported for various soil microorganisms; however, their concentrations and uniqueness varies among the species (Tahir et al., 2017; Cordovez et al., 2018).

### Siderophore Production

Iron deficiency is the major limiting factor causing chlorosis in plants, and it ultimately affects crop quality and yield. The use of synthetic chelates to overcome the deficiency is not feasible mostly because of their poor biodegradability (Ferreira et al., 2019). Siderophores, minor organic molecules produced by microorganisms and a few gramineous plants under iron-deficient conditions, enable the plants to uptake iron from the surrounding environment even in reduced iron availability (Saha et al., 2016; Prabhakar, 2020). They are important compounds for phytostabilization under unfavorable circumstances and provide metal coalescence, improve plant growth, and reduce metal bioavailability in the soil (Qessaoui et al., 2019). Research on siderophores during the previous decade has demonstrated their ability to extract iron ions (Saha et al., 2016; Kumar et al., 2018). PGPR, such as *Pseudomonas* sp., uses the siderophores produced by other microbes in the rhizosphere to meet their essential ion requirements (Qessaoui et al., 2019). Similarly, *P. putida* has been reported to accumulate and use heterologous siderophores produced by other microorganisms to overcome their iron deficiency by increasing the level of iron offered in the natural habitat (Gouda et al., 2018). The ferric-siderophore complex, an extremely strong siderophore, plays a vital part in the uptake of iron by plants in the presence of other metals, such as nickel and cadmium (Beneduzi et al., 2012). Research on siderophores and their capability to enhance the iron uptake ability of plants is still inadequate, and extensive studies are required to understand their behavior and mode of action (Prabhakar, 2020). Consequently, finding environment-friendly and appropriate siderophores with precise action, as well as usability as iron enrichers, is a challenge. Among various compounds, siderophores are receiving greater attention because of their role as iron chelators and the positive characteristic of biodegradability over synthetic APCAs (Fazary et al., 2016).

Three bacterial species, *Bacillus megaterium*, *B. subtilis*, and *A. vinelandii* expressed the maximum iron-chelating capacity, suggesting their potential to help overcome the iron deficiency in plants (Ferreira et al., 2019). Recent research described synthetic compounds, including catecholate and hydroxamate groups, as probable iron-chelating compounds that can provide nourishment and growth to plants (Martins et al., 2018; Ferreira et al., 2019). The use of siderophores in agriculture is practically limited because of their complex structure and difficulty to produce owing to a multistep but low yielding process (Leydier et al., 2008; Martins et al., 2018; Table 8).

**TABLE 8:** Plant growth-promoting rhizobacteria and siderophore production under drought stress.

Soil microbe/Strain	Plant species	Effect under drought	References
*Bacillus sp.* KB122, KB129, KB133, and KB14	Sorghum	Production of siderophore IAA and solubilization of phosphate.	Grover et al., 2021
*A. chroococcum* 67B	Tomato	Siderophore synthesis, N_2_-fixing activity	Viscardi et al., 2016
*B. phytofirmans* PsJN	Arabidopsis	Biosynthesis and transport of siderophore genes	Zhao et al., 2016
*Bacillus amyloliquefaciens* FZB42	Arabidopsis	Effect the formation of biofilm under drought	Lu et al., 2018
O. pseudogrignonense RJ12, *Pseudomonas sp.* RJ15, *B. subtilis* RJ46	Black gram	Synthesis of siderophore and phosphate solubilization	Saikia et al., 2018b
*A. aneurinilyticus* WBC1, *Aeromonas* sp. WBC4, *Pseudomonas* sp. WBC10	Wheat	Production of siderophore	Kumar et al., 2018
*Pseudomonas* sp. Q6B, Q14B, Q7B, Q1B, and Q13B	Tomato	Phosphate solubilization, production of ammonia and siderophore	Qessaoui et al., 2019
*Azotobacter sp.* Az63, Az69, and Az70	Maize	Enhanced siderophore production along with phosphate and potassium solubilization	Shirinbayan et al., 2019
*Bacillus amyloliquefaciens* 54	Tomato	Enhanced the biofilm-forming ability	Wang et al., 2019
*V. paradoxus* RAA3, *O. anthropi* DPC9, *Pseudomonas sp.* DPB13 *Pseudomonas sp.* DPB15 *Pseudomonas sp.* DPB16	Wheat	Synthesis of siderophore and phosphate solubilization	Chandra et al., 2019
*P. aeruginosa* JHA6 *Bacillus amyloliquefaciens* ROH14	Pepper	Synthesis of siderophore, ACC deaminase activity and IAA production.	Gupta and Pandey, 2019
*Rhizobacteria sp*. AV-1, AV-2, and AV-7	Pulses	Siderophore production	Andy et al., 2020

### Transcriptional Response of Plant Growth-Promoting Rhizobacteria to Drought Stress

Gene expression studies are useful to understand and compare the responses of an organism to its environment (Azeem et al., 2018). Gene expression under drought stress was recently characterized using molecular approaches, and their physiological roles were studied with respect to tolerance induced by PGPR (Ghosh et al., 2019). At the transcriptional level, PGPR-enhanced plant tolerance to drought was observed after inoculation with *P. polymyxa* B2, with enhanced drought tolerance in *Arabidopsis thaliana* (Timmusk et al., 2014). RNA display revealed that the mRNA transcription of a drought-response gene *ERD15* was augmented as an early response to dehydration in inoculated plants compared with that in non-inoculated plants (Timmusk et al., 2014). Using two-dimensional polyacrylamide gel electrophoresis and differential display polymerase chain reaction, six differentially expressed stress proteins were identified in pepper plants inoculated with *B. licheniformis* K11 under drought stress. Among them, drought-specific genes *sHSP* and *CaPR-10* exhibited a greater than 1.5-fold increase in treated plants compared with that in control plants (Lim et al., 2013). Using real-time PCR, upregulation of stress-related genes *apx-1*, *sams-1*, and *hsp* 17.8 in wheat leaves and increased activity of enzymes involved in the plant ascorbate glutathione redox cycle, conferring drought tolerance in wheat, were identified when primed with *Bacillus amyloliquefaciens* 5113 and *A. brasilense* NO40 (Kasim et al., 2013). Using microarray analysis, a set of drought-signaling response genes were downregulated in the *Pseudomonas chlororaphis* O6-colonized *A. thaliana* compared with those without bacterial treatment under drought stress. Although the transcripts of the JA-marker genes *vsp-1* and *pdf-1.2*, SA regulated gene *PR-1*, and ET-response gene *HEL*, were upregulated in colonized plants, they differed in their responsiveness to drought stress (Cho et al., 2013). PGPR contains several functional genes, such as IAA production (*iaaM*), nitrogen fixation (*nifU*), spermidine (*speB*), and siderophore (*sbnA*) biosynthesis, which facilitate plant growth and tolerance under stress conditions (Xiong et al., 2019; Table 9).

**TABLE 9:** Upregulation of stress-responsive genes by PGPR under drought conditions.

Soil microbe/Strain	Plant species	Effect under drought	References
*Bacillus amyloliquefaciens* 5113	Wheat	Upregulation of stress related genes *APX1*, *SAMS1*, and *HSP17.8*	Kasim et al., 2013
*P. chloroaphis* O6	Arabidopsis	Transcription of JA biosynthesis (*VSP1, pdf-1.2*) and salicylic acid regulated gene (*PR-1*)	Cho et al., 2013
*Bacillus licheniformis* K11	Pepper	Inoculation increased the expression of stress responsive genes *Cadhn, VA, sHSP*, *and CaPR-10*	Lim and Kim, 2013
*Azospirillum brasilense* SP-7, *H. seropedicae* Z-152	Maize	Upregulation of ABA biosynthesis gene *ZmVP14*	Curá et al., 2017
*P. putida*	Chickpea	Activation of ethylene, salicylic acid (*PR1*) and jasmonate (*MYC2*) biosynthesis genes under drought	Tiwari et al., 2016
*B. subtilis* LDR2	Wheat	Upregulate the expression of *TaCTR1/TaDREB2* TFs under drought stress	Barnawal et al., 2017
*P. flourescens* Pf1	Rice	The activations of ABA mediated signaling pathway genes like *bZIP1, AP2-EREBP*, and *Hsp20*	Saakre et al., 2017
*B. subtilis* strain SYST2	Tomato	Enhanced the expression of auxin (*SlIAA1. SlIAA3*), gibberellin (*GA20ox-1*), CK (*SlCKX1*), expansion (*Exp2, Exp9. Exp 18*), and ethylene (*ACO1*) biosynthesis genes	Tahir et al., 2017
*Bacillus amyloliquefaciens* FZB42	Arabidopsis	Expression of drought defense related genes such as *RD29A, RD17, ERD1*, and *LEA14*	Lu et al., 2018
*Bacillus amyloliquefaciens*	Tomato	Elevated expression of stress responsive genes, i.e., *lea*, *tdi65*, and *ltpg2*, increased in	Wang et al., 2019
*Streptomyces sp.*	Tomato	Modulate the expression of TF *ERF1* and *WRKY70* under drought stress	Abbasi et al., 2020
*F. crocinum* HYN0056	Arabidopsis	Upregulation of drought responsive genes *RD29A* and *RAB18*	Kim et al., 2020
*Trichoderma sp.* *Pseudomonas sp.*	Rice	Over expression of water permeability (*OSPiP*), drought adaptation (*DHN*) and dehuderation genes (*DREB*)	Singh et al., 2020

### Interactive Effect of Drought and Other Stresses

Drought and other abiotic stresses, including salinity, temperature extremes, biotic stress, and malnutrition, mostly occur simultaneously. The combination of drought and other stresses causes a severe inhibition of physiochemical activities and growth in food crops. For example, plants demonstrate identical physiochemical and morphological symptoms when subjected to drought and salt stress (Ahluwalia et al., 2021). Higher salt concentration favors the occurrence of drought stress because salt-related solutes reduce the uptake of water, resulting in reduced leaf water content (Sagar et al., 2022). Plants in association with PGPR alleviate salt stress by improving their antioxidative machinery, reducing the level of lipid peroxidation and ROS, enhancing the synthesis of biomolecules and phytohormones, regulating osmosis, and increasing gas exchange attributes (Nadeem et al., 2010; Akram et al., 2019). Similarly, halotolerant PGPR modulates gene expression and osmolyte production to improve salinity tolerance and growth in *Capsicum annum* (Yasin et al., 2018a; Khan M. A. et al., 2019; Sagar et al., 2022).

The joint stress caused by heat and drought in arid, semiarid, and tropical regions reduces photosynthetic activity, stomatal conductance, and CO_2_ assimilation in plants. The interactive effect of heat and drought stress reduces RuBisCO, photosystem II, and chlorophyll biosynthesis activities while enhancing the foliage temperature (Raja et al., 2020). It was observed that the synergistic effect of heat and drought stress restricted the development of pollen, pistil, and ovule in grain crops (Ahmad et al., 2021). The increased synthesis of ROS in plants subjected to heat and drought stress denatures the proteins, declines plant nutrition, reduces membranous stability, and deteriorates the antioxidant defense system, leading to decreased growth and biomass production in crop plants (Ahluwalia et al., 2021). However, the increased synthesis of osmoregulators and improvement in the antioxidative system because of PGPR assisted the stressed plants to enhance their tolerance by reducing the level of MDA, ROS, and other toxic elements that may decrease plant growth (Shah et al., 2021a; Tariq et al., 2021).

Drought may enhance the chances of pathogenic attack and infection in crop plants. Drought-stressed plants will close their stomata to reduce water loss through transpiration. Nevertheless, pathogen-infected plants enhance their rate of transpiration (Aung et al., 2018). The toxins produced by *Uromyces phaseoli*, which causes leaf rust in *R. phaseoli*, decrease the stomatal openings, leading to conciliated drought resistance (Duniway, 1976). Although a gentle drought triggers the plant defense system to reduce the pathogen infection, severe drought causes enhanced pathogen virulence because plant cells discharge nutritious compounds on their apoplast, which supports the growth and pathogenicity of the plant pathogens (Ahmad et al., 2020; Singh et al., 2020). Wheat plants infected by *Fusarium culmorum*, which causes seedling blight and root rot disease in wheat, exhibited reduced plant growth and biomass production owing to enhanced levels of MDA content under drought stress regimes (Lastochkina et al., 2020). Several PGPR strains trigger the defense systems of plants to combat diseases. Inoculation with *Bacillus* and *Pseudomonas* bacterial strains may induce disease resistance in crop plants through the modulation of antioxidant enzymes and osmoregulators (Yasin and Ahmed, 2016).

Plants growing in areas with metal pollution exhibit curtailed routine physiochemical and molecular activities (Yasin et al., 2018b; Shah et al., 2021b). The interactive effect of drought and metal stress imposes highly pronounced negative effects on the physiology, morphology, growth, and yield of crop plants (Yasin et al., 2018c). However, several PGPR strains are capable of mitigating metal toxicity. *Catharanthus roseus* plants inoculated with *Bela fortis* 162 exhibited improved root and shoot growth in addition to oxidative stress tolerance under chromium exposure (Yasin et al., 2018d). Similarly, *P. fluorescens* RB4 and *B. subtilis* 189 mitigated the combined stress induced by Cu and Pb in assisted plants (Khan et al., 2017a). Inoculation with *Bacillus* spp. and *B. megaterium* MCR-8 in plants growing under nickel stress improved their antioxidative potential and gas exchange attributes (Khan et al., 2017b). In addition to the individual effect of PGPR in stress alleviation, these microbes may enhance the efficacy of exogenously applied stress ameliorants, including nanoparticles, plant nutrients, and phytohormones. The interaction of *B. subtilis* FBL-10 and silicon reduced the effect of lead toxicity in eggplant (Shah et al., 2021b). The synergistic effect of iron oxide nanoparticles and *B. subtilis* S4 alleviated arsenic toxicity in *Cucurbita moschata* (Mushtaq et al., 2020). Application of *Bradyrhizobium japonicum* EI09 and selenium improved chromium stress tolerance in *C. annum* (Nemat et al., 2020). Similarly, *B. thuringiensis* IAGS 199 and putrescine alleviated cadmium-induced phytotoxicity in *C. annum* (Shah et al., 2020). Furthermore, synergism between *Enterobacter* sp. CS2 and ethylenediaminetetraacetic acid exhibited positive effects on the growth of plants subjected to Ni stress (Yasin et al., 2018d).

## Conclusion

Thus, drought stress not only affects the morphological and physiological characteristics of plants, leading to a loss in crop production but also affects the soil microbe interactions. We discussed the ways that PGPR adopt to enhance drought stress resistance. Soil microorganisms associated with the root system of a plant change the cell membrane elasticity of the roots, which eventually increases the drought tolerance capacity. However, during drought stress conditions, plant growth can be improved by the rhizosphere microbial community *via* an increase in the root surface area and root production. We also enumerated various crop data to demonstrate the way PGPR are involved in managing the metabolic changes, EPS production, 1-aminocyclopropane-1-carboxylate deaminase activity, phytohormone production, antioxidant activities, ROS accumulation, siderophore production, and transcriptional response to drought stress.

## Author Contributions

HA conceived the idea and collected a literature review. SH, SZ, OA, and Mahmood-Ur-Rahman helped in the original draft. AS, XW, and YC critically reviewed the initial draft and streamlined the idea. MR, BG, and AF prepared and revised the figures. SY and SF helped in funding acquisition and revision of the manuscript. All authors carefully read, revised, and approved the manuscript for submission.

## Conflict of Interest

The authors declare that the research was conducted in the absence of any commercial or financial relationships that could be construed as a potential conflict of interest.

## Publisher’s Note

All claims expressed in this article are solely those of the authors and do not necessarily represent those of their affiliated organizations, or those of the publisher, the editors and the reviewers. Any product that may be evaluated in this article, or claim that may be made by its manufacturer, is not guaranteed or endorsed by the publisher.
